# Uncovering hidden abilities for participation in research through photo-elicitation interviews: a view on participatory research with people living in residential care facilities

**DOI:** 10.1186/s40900-023-00422-9

**Published:** 2023-03-18

**Authors:** Qarin Lood, Roar Hermansen Østby, Sara Hultqvist, David Edvardsson, Synneve Dahlin-Ivanoff

**Affiliations:** 1 Department of Health and Rehabilitation, Institute of Neuroscience and Physiology, Sahlgrenska Academy, Centre for Ageing and Health –AgeCap, University of Gothenburg, Box 455, 40530 Gothenburg, Sweden; 2 School of Nursing and Midwifery, Faculty of Health Sciences, La Trobe University, GS Building, Office 327, Bundoora, 3083 Australia; 3 Faculty of Social Science, Department of Social Work, Linnaeus University, 351 95 Växjö, Sweden; 4 Faculty of Medicine, Department of Health Sciences, Lund University, Lund, Sweden; 5 Centre for Person‑Centred Care (GPCC), University of Gothenburg, Gothenburg, Sweden; 6 Sahlgrenska Academy, Institute of Health and Care Sciences, University of Gothenburg, Gothenburg, Sweden; 7 Institute of Neuroscience and Physiology, Department of Psychiatry and Neurochemistry, Sahlgrenska Academy, University of Gothenburg, Blå Stråket 15, Vån 3 SU/Sahlgrenska, 413 45 Gothenburg, Sweden

**Keywords:** Participatory research, Photo elicitation, Residential aged care, Nursing homes, Care homes, Qualitative methods, Methodology, Frailty, Dementia

## Abstract

**Background:**

Participatory research has been described to improve the relevance of research findings for the society in terms of quality of healthcare services and other public benefits. Nevertheless, there is limited guidance on how to conduct participatory research, and especially in relation to persons living in residential care facilities. To make the voices of this group heard, we therefore take a stance in the democratic approach to participatory research, and we have applied the theoretical framework Model of Human Occupation (MoHO) on participation to evaluate photo-elicitation interviews as a participatory research method with this group.

**Methods:**

A total of 13 persons living in two residential care facilities were involved in the study and asked to take photographs of their everyday life over one week. They were then invited to an individual interview to narrate the meaning of the photographs and to describe how they experienced the photo-elicitation method. The interviews were analysed in the six steps of theoretically driven reflexive thematic analysis.

**Results:**

The findings are described in the theme ‘Uncovering hidden abilities for participation in research’ that describes how photo elicitation interviews facilitated the older persons’ participation in research. This is illustrated by four sub-themes: ‘Bridging the ageing body’, ‘Altering habituation to everyday life’, ‘Empowering storytelling’, and ‘Negotiating the institutional culture’.

**Conclusions:**

Our study findings support further application and evaluation of photo-elicitation interviews as a method for participatory research in residential care facilities. The major finding is how photo-elicitation interviews were used to reduce the impact of the institutional culture on the older persons’ participation in research. The method is, however, not without limitations and we encourage researchers to study the dynamic relationship between physical, social, and cultural aspects of residential care facilities in relation to the use of photo-elicitation interviews with the persons living there.

## Abstract (summary)

This study was designed to improve the opportunities for persons living in residential care facilities to participate in research on their own terms. Digital instant print cameras were used by 13 older persons to document their everyday life at two different residential care facilities. The photographs were then followed up with individual interviews, during which the older persons were asked to share their experiences of using the cameras and to tell stories about the meaning of the photographs. Analysing the interviews, we found that the older persons could participate in the study in a way that they did not believe was possible before. They were not used to being given responsibility, and they felt limited by physical and cognitive impairment. Participating in the study, they realised that they had something to contribute with, and that there were aspects of everyday life that they really enjoyed. They felt empowered to share their stories, and we encourage researchers to continue evaluating photographs in combination with interviews in residential care facilities.

## Background

Based on the notion that people outside academia are experts on their experiences and know best what is needed to improve their lives, this study was designed to evaluate photo-elicitation interviews as a participatory research method with people living in residential care facilities. In Sweden, persons who live in residential care facilities are generally affected by both frailty and cognitive decline, which influences their functional ability and contributes to a dependence on other people in everyday life [1]. Moreover, previous research visualises how residential care facilities are directed by staff and that the persons living there have few opportunities to influence their everyday life or choose what activities to be involved in [2, 3]. In relation to participatory research, this might mean that they are never even asked if they want to participate in research.

According to the World Health Organization (WHO)[4], participation is defined as “involvement in a life situation” [4], and they describe how body function and structures, environment and personal factors, activities and participation all interact with each other dynamically. They further describe how participation is both a subjective and objective phenomenon, as it incorporates a person’s engagement, opportunities to feel accepted, and access to necessary resources [4]. Inasmuch as participation in research has been described, there is an array of definitions, methodologies, methods, and incentives for participatory research [5, 6]. Concepts such as user-driven research, community based participatory research, co-design, participatory design, co-production of knowledge, patient and public involvement, patient-driven research, participatory action research, and collaborative research are all being used to describe participatory research approaches [7], which contributes to a conceptual blurring of the phenomenon. For the aim of this study, participatory research is the concept used to describe research conducted together with people rather than on or for people. As described by Cornwall and Jewkes [8], the main difference between participatory research and other research approaches is the distribution of power between the researchers and the persons who are participating in the study [8]. This means that we draw on a democratic perspective on the research process as the motif for participatory research [9], to distribute power more equally between researchers and people living in residential care facilities.

The democratic perspective on research is built on the understanding that participatory research aims to strengthen a group’s opportunities to make their voices heard, involving a striving towards shifting the power, from researchers to the persons involved in the research as research persons [10]. As there is a long history of excluding older persons from research [11, 12] we apply the democratic perspective on participatory research, to acknowledge persons living in residential care facilities as capable and holding the same rights to make their voices heard as anybody else. By involving the persons who are likely to be affected by the research conducted, the relevance of research findings for society is expected to increase [13]. Yet, as indicated above, recommendations for how to apply participatory research approaches vary. Some recommend participatory approaches at all stages of the research process, from generation of research questions to presentation and dissemination of research findings [14], while others propose that participatory research operates along a continuum with pros and cons at each level of participation [15]. A main challenge with participatory research can thus be derived to the difficulties in understanding what participation in research means and what is expected from each party in the participatory process [16].

Participatory research implicates collaboration between researchers and actors outside academia to provide diverse perspectives on a research topic. As such, participatory research approaches depend heavily on building trust and nurturing close collaborations between researchers and actors outside academia [8] to make use of both scientific knowledge and the insider knowledge that comes from lived experiences [17–20]. Defined by collaboration, participatory research is argued to produce high quality healthcare services [21] as well as academic insights and public benefits [22]. It has been described as research that strives for shared ownership between researchers and actors outside academia, with the overarching goal to co-create research [19]. Beyond this, there is little guidance on the practicalities of participatory research [23, 24]. There are also limited explorations on what might influence participatory research with different people in different contexts. For instance, older people living with frailty have rarely been invited to participatory research projects [25], and when they have been invited, they have primarily been used as sources of data to develop professionally designed services and outcomes, rather than being involved as equal partners with valuable knowledge beyond the expertise of the researchers [26]. One explanation to this, raised in previous literature, is negative presumptions about the abilities of this group to contribute to the research process, with perceived difficulties with access to research and communication between them and the researchers [27, 28]. This raises serious concerns with regards to both the transferability of findings, and to the opportunities for older people living with frailty to make their voices heard, to influence research, and to make use of research findings. As described by Berge et al. [28] research could be understood as someone else’s home turf, and older people living with frailty might be in doubt regarding their role in the research project [28]. Moreover, there might be a need for adapted strategies for participatory research approaches with people experiencing cognitive decline [29], who are often excluded from research due to supposed difficulties associated with their participation or the belief that there would be limited benefits with their participation [30]. In previous qualitative research with people experiencing cognitive impairment, semi-structured interviews have been used to explore their experiences [31, 32], sometimes with adaptations made to the interview technique [29, 33]. Nevertheless, cognitive impairment affects the ability to recall and report on experiences verbally, which may have a negative impact on the opportunity and ability to participate in qualitative interviews [34]. Drawing on the literature on visual research methods, we therefore sought for a method that would allow people living with cognitive impairment to participate in research on their terms. In particular we were interested in exploring what might affect participation in research that incorporates visual methods among people living in residential care facilities.

Visual research methods may include photography, video, or artwork [35] to bring another dimension to the research data than conversations alone [36]. Visual research methods can also provide valuable insights into the life of the persons involved in the study [35] that cannot be captured by verbal methods. Pain [37] illustrates this in a literature review, describing how visual research methods could be used to facilitate dialogue, enhance rapport building, encourage reflection, and enable the expression of unspoken or unexpressed aspects of the studied phenomenon. In this study, photo-elicitation interviews were chosen based on their potential to evoke feelings, memories, and information on subjective experiences. Photo-elicitation interviews refer to the idea of using photographs in research interviews to explore a certain topic and the photographs can be generated by either the researcher or the research person [38]. The method has recently been used to involve older people in research [39, 40] but as highlighted in a review of the photovoice method with older persons [41], visual research methods can be practically challenging due to ethical issues, functional and visual impairment, logistics, and resources allocated to the research project. The review further suggests that visual methods need accommodation to different contexts [41], but there has been little attention to the potential benefits of photo-elicitation interviews as a participatory research method with people living in residential aged care facilities. Therefore, we set out to evaluate photo-elicitation interviews in relation to facilitators and limitations for participation in research among this group. To operationalise participation, we applied the Model of Human Occupation (MoHO) [42], which is a systems theory that describes how people interact with the environment when conducting an activity. According to MoHO [42], a person’s participation in an activity (such as a research activity) can be understood as collectively influenced by their: performance capacities, habituation, volition, and environmental conditions. Performance capacities are defined as a person’s physical and mental abilities to perform an activity and concern both objective depictions and subjective experiences of those abilities. Habituation refers to the way people organise their activities into patterns or routines, and the things people do unreflectively because they are so familiar that they are taken for granted. Volition denotes a person’s motivation to do a certain activity, which is influenced by their sense of competence and effectiveness, as well as their values and interests. Environmental conditions comprise physical, social, and cultural features of the context within which people live and act [42].

## Methods

With the aim to evaluate photo-elicitation interviews as a participatory research method with persons living in residential care facilities, the MoHO was used as a theoretical tool to identify what could influence participation. More specifically, MoHO guided the evaluation through the following research questions:

- How do the older persons’ performance capacities, habituation, and volition influence participation in research through photo-elicitation interviews?
- How do environmental conditions of a residential care facility influence older persons’ participation in research through photo-elicitation interviews?

A total of 13 persons living in two Swedish residential care facilities were asked to use instant print digital cameras to document their everyday life in the facility. The photographs were then followed up by individual interviews. Given the focus of this study, the content and meaning of the photographs will not be overly elaborated on. This will be reported in a separate publication.

### Study context

The study was conducted in two residential care facilities in different suburbs in a mid-sized Swedish city. One of the facilities housed up to 102 persons with varying degrees of cognitive impairment, and the other facility had a primary focus on people diagnosed with dementia and housed up to 64 persons. The level of care and support provided to each person depended on professional assessments of their abilities [43], but in accordance with Swedish regulations for residential care facilities for older people, all persons had access to direct care staff (mainly assistant nurses with upper secondary care education) round-the-clock [1, 43]. Allied health professionals and physicians were also available depending on the needs of each person.

### Involvement

Managers and staff at each residential care facility acted as gatekeepers, assisting with involvement of eligible persons by assessing eligibility and distributing written and verbal information to eligible persons. Eligibility criteria were: 1) living in a residential care facility, 2) assessed by staff as cognitively able to give informed consent and 3) able to hold a conversation for at least 15 min. A total of 15 persons were assessed as eligible by staff and were presented with information on the study and what involvement would mean before being invited to be involved in the study. Two persons declined participation due to feelings of insecurity relating to having responsibility for a camera. Due to the COVID-19 pandemic, eight of the involved persons were invited by two staff members who knew the older persons but were not involved in their direct care. The remaining five persons were involved at a later stage of the pandemic, which made it possible for the first author to provide them with information and involve them in the study. All persons provided written informed consent before being involved in the study.

The 13 involved persons were between 71 and 94 years of age, eight lived in the larger facility, five in the smaller facility, 10 persons were women and three were men. All involved persons were assessed by the first author as living with frailty according to the FRESH screening tool [44], i.e., they answered yes to at least two of the following four questions: 1) “Do you get tired when taking a short (15–20 min) walk outside?”, 2) “Have you suffered any general fatigue or tiredness over the last three months?”, 3) “Have you fallen these last three months?” and “Are you afraid of falling?”, and 4) “Do you need assistance in either getting to the store, managing obstacles (such as staircases) to and from the store, or in choosing, paying for, or bringing home groceries?” [44].

### Instruction, photo period and individual interviews

Data were produced in two steps, between November 2020 and November 2021 to cover all seasons and their potential influence on the involved persons’ experiences. To build trust and rapport, the first author came to the first visit accompanied by a staff member that the older person knew well. First, each person was provided with an instant print digital camera, allowing for up to 10 photos to be taken by each person over a period of between two and seven days. The choice of camera was based on it being simple to use, with only two buttons: one for switching the camera on, and one for taking pictures. This meant that all persons with sufficient hand strength and dexterity could operate the camera. Moreover, the instant print function provided the older persons with the opportunity to see their photographs on the camera screen and choose what photographs to print either immediately after taking them, or when the researcher came for the interview.

All persons received instructions on how to use the camera and were instructed by either staff (for the first eight persons) or by the first author (for the remaining five persons) on how to use the camera. They also got the opportunity to practice using the camera during the involvement encounter. The older persons were encouraged to take photographs themselves, but upon request, staff familiar with the person and the facilities assisted the older persons by prompting them to take photographs and/or helping them handle the camera. This was the case for a majority of the involved persons, but everyone was able to decide what motifs to take photographs of.

All involved persons were asked to take photographs of situations, objects, places, and spaces within the residential care facility, documenting aspects of relevance for their experiences of their home, and they were encouraged to take photographs both inside and outside the facility. Then, the first author contacted them to schedule a time for a follow-up interview, which was conducted in the older persons’ apartments using medically approved protective equipment. During the interviews, the older persons were asked to 1) choose which photographs to print and talk about (the printed photographs were 75 × 50 mm), 2) contextualise each photograph, to ascribe meaning to the visual images, and 3) to elaborate on their experiences of using the camera and participating in the study. They were also encouraged to share their thoughts on experiences that they felt they could not document by photographs, and on reasons as to why that was not possible. The interviews had the character of a dialogue, and examples of interview questions were: “What do you remember about taking these photos?”, “What were your thoughts when taking the photo?”, “What does the motif mean to you?”, “How did you feel using the camera?”, “Was there anything you wanted to describe but could not describe using photographs?”. The older persons took between one and 10 photographs each (a total of 80 photographs) and selected up to five photographs to talk about during the interview.

Due to pandemic-related restrictions, with no visitors allowed to the residential care facilities, the first eight interviews were conducted approximately six weeks after the photographs had been taken. The remaining five interviews were conducted immediately after the photography period. All interviews were digitally recorded and transcribed verbatim by the first author who also took fieldnotes during recruitment and interviews and interviewed staff who had assisted with the photography. The intention with the fieldnotes was to provide a deeper understanding of the research setting and procedure than had been acquired through photography and interviews alone. Field notes included data on subjective and personal account of the researcher’s experiences of each stage of the data generation.

### Transcription and analysis

Due to the focus and purpose of this paper, the photographs were not systematically analysed for content and meaning but rather in terms of how photo-elicitation facilitated or obstructed the older persons’ participation in research. However, in general, the motifs were everyday objects such as mailboxes, coffee cups, pencils, and clocks, as well as images of perceived issues such as towels on the floor, poor lighting and alarms that did not work. A thorough analysis of the photographs and the older persons’ narrations on their meaning will be presented in a separate publication. The transcribed interviews and the fieldnotes were analysed using theoretically driven reflexive thematic analysis [45–47], involving the following six phases: 1) Familiarisation with data, 2) Deductive organisation of data onto the MoHO’s description of aspects related to activity participation, 3) Inductive search for themes, 4) Reviewing the themes, 5) Defining and naming themes, 6) Reporting the analysis. The analysis process started by transcription of the interviews, listening to all interviews and reading all transcriptions and fieldnotes repeatedly to get familiarised with the data. The second step involved the deductive organisation of the text using the MoHO [42] concepts performance capacities, habituation, volition, and environmental conditions. The analysis then proceeded to an inductive phase, allowing for interpretation of the deductively organised text. This involved a search for the latent meaning and implications of the extracted data, resulting in the formulation of prospective themes that were reviewed and revised. Then, all authors discussed the deductive organisation, prospective themes, and the transcribed data to define and name themes, organising them into a narrative structure. Considering internal homogeneity and external heterogeneity [48], this step also involved a refinement of the themes, and the data extracts for each theme were assessed regarding their fit within the theme or not. The validity of each theme was also considered in relation to the data set as a whole (including fieldnotes). Finally, the data extracts were analysed within each identified theme and final themes were defined based on the meaning and implications of the text. This step (step 5) continued until it was not considered possible to conduct any further refinements of the themes, and the themes were named and translated to English for the final production of the report which is described in the results section.

## Results

The analysis resulted in the overarching theme *Uncovering hidden abilities for participation in research* that describes how photo-elicitation interviews facilitated the older persons’ participation in research. This is described in four sub-themes that illustrates how individual performance capacities (*Bridging the ageing body)* habituation (*Altering habituation to everyday life)* and volition (*Empowering storytelling),* influenced participation together with the environmental conditions of the residential care facilities* (Negotiating the institutional culture)*. The theme and sub-themes are described in more detail below, contextualised by quotations from a selection of persons that have been given fictive names.

### Uncovering hidden abilities for participation in research

The overarching theme illustrates how photo-elicitation interviews could be used as a tool for co-creation of knowledge. Challenging the mindset of both staff and the older persons, the method facilitated the older persons’ participation in research by putting focus on the potential of the persons involved, rather than on their limitations. Abilities hidden by presumptions related to functional decline, cognitive impairment and everyday life in an institutional environment were uncovered and the photographs gave the older persons a different perspective on their opportunities to participate in research through storytelling and empowerment.

### Bridging the ageing body

This sub-theme describes how the photo-elicitation interviews helped the older persons bridge barriers to participation imposed by their ageing bodies. Affected by limited performance capacities, they felt that they could not do what they were expected to do in the study, described as being able to handle the camera independently and remembering to take photographs. Difficulties with understanding how the camera worked and with moving around independently to take the photographs were bridged by having staff around to assist them, which meant that the residential care facility became a facilitator for participation. Barriers imposed by limited performance capacities were also bridged by the researcher who assisted the older persons during the interview by describing what was on the photograph. This allowed for narratives on what the photograph was supposed to mediate even if the older persons had little or no recollection of using the camera regardless of whether they used it the day before the interview or several weeks before the interview. The photographs sparked the older persons’ memory and made it possible for them to describe what specific people, objects or situations depicted in the photographs meant to them. We have chosen a quotation from Mona as an example:

Interviewer: Then you have taken a few photographs and printed them. There are five photographs. Do you remember them?

Mona: No

Interviewer: Let’s see, they are pretty small.

Mona: Well, yes reasons for me taking them. It is our store.

Interviewer: Ah, is it a store?

Mona: And it, it it is almost just a shadow of a store.

Interviewer: And what do you have in the store?

Mona: Sweets and laundry powder, cream and shavers and lamps and…

Interviewer: Ah, ok. And what was the reason for you taking (the photograph)

Mona: It was because we should have an ATM close to it.

Interviewer: You would want that?

Mona: Mmm. Now I am completely dazzled, in that I have not. I cannot go to, I cannot buy anything because I have no cash left.

Altering habituation to everyday life

#### Altering habituation to everyday life

In this sub-theme, the photo-elicitation interviews are described in relation to how they gave the older persons a new perspective on everyday life at the residential care facility. Challenging the commonplace experiences of the residential care facility as dull and mundane, altering habituation to everyday life meant that the photographs told their own stories on the beauty of everyday life. Even when the older persons struggled to find things interesting enough to share with the researcher, the photographs helped them realise that there were aspects of the facility that they really appreciated, and the opportunity to keep the photographs after the interview was valued highly. For instance, photographs of flowers or the view from a window told stories about the beauty that existed around them, hidden behind the curtains of everyday life. A such, the photo elicitation interviews bridged barriers for participation by providing the older persons an opportunity to see and narrate details they did not previously think about as interesting. This altered habituation to everyday life is visualised by the following quotation from Sonja, when answering a question about her participation in the study.

Interviewer: Good, how has it been to use the camera? How has it been for you to use this? Has it worked?

Sonja: Well, I think it has been a lot of forgetfulness (laughs a little). And, ehm, in a place like this there is not much to document either.

Interviewer: No, what are your thoughts on that?

Sonja: Yes, at it might be connected to that this is, has been my everyday life.

Interviewer: That’s right.

Sonja: So that you, ehm, don’t see anything particular in it. This is not so, it has always been like this and so on. I don’t know if it is that which has, ehm, affected it a little bit.

Interviewer: Let’s see here, here we have?

Sonja: Yes, this is the view.

Interviewer: From your room here?

Sonja: And, ehm, the memories from here below.

Empowering storytelling

#### Empowering storytelling

The photographs used during the interviews stimulated the older persons’ curiosity and volition to share their experiences with someone who listened to what they had to say. Feeling acknowledged by the researcher, the older persons chose to discuss certain photographs and told stories about what the motifs meant to them, even if they had not been able to take the photographs independently. To make their stories complete, they also used diaries and notebooks to document aspects that could not be captured by photographs either due to the sensitivity of asking staff to take photographs of issues related to care and services at the facility, or to a lack of access to the camera when realising something that they wanted to share. This was described by Agata in the following quotation:

Agata: I will have a look into what I have written here you see.

Interviewer: Yes, you keep a diary there?

Agata: Yes, Well, our front door. They lock it at four o’ clock.

Interviewer: Yes, I saw the sign there yes.

Agata: And that I would think that they should change it to five o’ clock. Because in summer, now it is nothing. But during summer, when everyone sits outside. Then there are those who, it is rarely windy by the front door, so they sit there.

### Negotiating the institutional culture

This theme visualises how the photo-elicitation interviews challenged institutional views of what was normally expected from the older persons as residents. The institutional culture was narrated as staff being in charge, with little or few opportunities for the older persons to realise their abilities and take charge over what they do. Thus, the embodied experience of being a resident initially resulted in the older persons not feeling confident in their abilities to participate in research, and they were worried about breaking or misplacing the camera. When talking to the researcher, the older persons did, however, feel empowered by being invited to participate in the study despite their experienced disabilities. Through their participation, they felt recognised as capable persons and took the opportunity to make their voices heard in a way that they were not used to. The researcher’s experience in healthcare (registered occupational therapist) was further described as a facilitator for establishing rapport and for sharing their stories with someone who they felt understood them and their situation. The following quotation from the interview with Leif was chosen as an example.

Interviewer: Is there anything you would like to add about this, how it was to have the camera for a few days and take pictures and…

Leif: I think it has been very fun. And I think it is, I think it is very fun that you are interested in what we think. Now you do research and such, but it is like, for me it was hard, or what should I say, not hard, but what was a little bit difficult when I moved to this residential care facility was being seen as demented, and what should we say, ehm, unintelligent.

Interviewer: That’s right

Leif: And it, I think it is just that, we say like this, we have a lot of knowledge. We have a lot of experience, so I don’t think it is right to judge us.

## Discussion

This study set out to evaluate photo-elicitation interviews as a participatory research method with persons living in residential care facilities. The main finding is that the method facilitated participation through providing the older persons an opportunity to make their voice heard and be listened to. Influenced by needs caused by actual impairments as well as perceived needs, not necessarily based in incapacity, the older persons participated in a way that they did not believe was possible before being involved in the study. The impact of the institutional culture on participatory research in residential care facilities as illustrated in our findings is supported by theories on the infantilisation of older people [49]. Thus, a key challenge for participatory research in residential care facilities is to develop dialogues between all people involved to allow for diverging perceptions. With the aim to provide people living in residential care facilities with responsibility over the data production, our study contributed to an awareness of their abilities to contribute to the research process. As such, photo-elicitation interviews could be an important tool for participatory research, facilitating co-creation of knowledge, empowerment, and change. Moreover, the method could be used to reveal the potential of older people living with frailty in a way that other research approaches cannot. It is, however, important to consider the social norms which govern the everyday life in residential care facilities, which may have a negative impact on older persons’ views on their abilities to participate in research. With documented difficulties for people living in residential care facilities to make their voices heard and influence daily routines and care processes [2, 3], participatory research with this group requires specific attention to how they interpret, understand, and narrate their experiences and needs. Indeed, ageing and frailty may have a negative influence on people’s opportunities to participate in research, but there is also important knowledge to be found if older persons living with frailty are given opportunities to participate in research on their terms [28, 50].

An important argument for our study was that photo-elicitation interviews do not rely on the ability to express oneself in words alone. Yet, our findings showed that the photographs were not primarily used to replace words, but rather to empower storytelling. As visualised in our findings, the photo-elicitation interview method helped uncover abilities that had previously been hidden by the older persons’ limited performance capacities and the institutional culture. Based on our findings, it is reasonable to believe that the photo-elicitation interviews allowed the older persons to share stories that they would not have been able to share in words-alone interviews. Harper [51] has described this as being a result of how people tend to respond to pictures compared with words. Thus, even if it was not possible for us to compare photo-elicitation interviews with interviews without photographs as prompts, we draw the conclusion that the photographs provided an important stimulus for the older persons’ narrations by helping them remember what they considered important. Our findings also support previous research on using photographs in interviews [52], highlighting the freedom to provide the people involved with power over the interview by being able to control what is being discussed.

Visual methods, such as photo-elicitation interviews, have the capacity to facilitate recollections and storytelling [53], but it is important to keep in mind that the older persons’ recollections might have been, at least partly affected by staff assisting the older persons with taking photographs. The support from staff might have had an impact on the extent to which the study was participant-driven and might have resulted in a distorted image of everyday life compared to how the older persons themselves would have depicted it. Nevertheess, our findings also portray the institutional setting as a facilitator for photo-elicitation interviews since staff were available to remind and assist the older persons with the camera. Without staff around the older persons might have forgotten about the camera completely, or not understanding how to use it. It is also important to emphasise that during the interviews, the older persons talked freely about aspects of everyday life that were not captured on photographs. The photo-elicitation method provided them with opportunities to overcome barriers relating to performance capacities and environmental conditions. However, even if the photographs and the interviews that followed provide insight into important aspects of everyday life for the persons involved in the study, the extent to which the methodology is to be regarded as a participatory approach in residential care settings requires further explorations.

## Methodological limitations

A strength of the method visualised in our study was that it captured everyday life experiences that the older persons might not have been able to remember or even see without the use of photographs. The combination of photographs and individual interviews to follow up the meaning and significance attributed to the images by the older persons proved to be an essential aspect of the study design as the photographs supported the older persons’ memory and helped them articulate what they considered being important. Barriers relating to impaired cognition were partly overcome by the researcher asking questions about the photographs, but in hindsight it would have been valuable to ask the older persons to write a statement on the meaning of each photograph immediately after taking it.

A limitation with our study is the use of staff as gatekeepers in the involvement of older persons, which might have resulted in an indirect exclusion of eligible persons. As such, we cannot guarantee that the older persons involved were representative of the overall group living in the facilities. This is especially important to consider in relation to the findings on how staff and older persons alike did not trust the older persons’ abilities to participate in research. Yet, as described by Sixsmith et al. [54], trust and rapport are essential to a successful recruitment. Thus, the use of pre-existing relationships between staff and the older persons were considered essential for us to encounter older persons interested in being involved. Another limitation relating to the involvement of older persons is the COVID-19 pandemic related access restrictions to residential care facilities. This meant that we were dependent on staff to select and ask the older persons if they wanted to be involved, which is likely to have led to selection bias. Especially since there were only 15 out of 166 persons assessed by staff as eligible for being involved. At the same time, Novek et al. [55] describe selection bias as a general issue in photo studies, indicating that it was not the pandemic related restrictions that led to this limitation. We kept close contact with staff responsible for involving the older persons, striving to ensure that they asked all persons who fulfilled our inclusion criteria. Nevertheless, the low number of eligible persons identified may be an indication that there were persons who were wrongfully excluded from being involved in the study, due to staff assumptions of them being incapable. As this was one of the issues that we wanted to address with our study, we encourage researchers to attend to issues with using gatekeepers for involvement of persons living in residential care facilities.

The COVID-19 pandemic also had an impact on the choice of study design, in that our initial plan was to conduct a photovoice study as described by Wang and Burris [56]. Due to the access restrictions and restrictions with physical distancing among people living in the facilities, we could not conduct focus groups to discuss the photographs as required by that method. We as researchers had limited access to the facilities during the study period, and it was not possible to involve external collaborators to facilitate community change which is an important part of the photovoice method. A strength with photo-elicitation interviews in our study was that it allowed us to capture individual experiences of the facility, as compared to photovoice which according to Wang and Burris [56] aims to capture group narratives to foster community change. Due to the change in methodology, our focus was never to implement change, but to explore democratic aspects of participatory research to give voice to persons whose voices are seldom heard [9].

There are some ethical issues to consider when planning and conducting photo-elicitation interviews in collective environments such as residential care facilities. For instance, the benefits of including photographs need to be balanced with the protection of privacy, both for the people directly involved in the study and for people in their surroundings that they might take photographs of. We were careful to talk to all people involved about the ethics on taking photographs of other people and ensured them that no photographs depicting themselves or other people would be published in any form. Another ethical issue relating to the institutional culture is the risk of withholding criticism. Although this might have been the case in our study, the older persons did speak freely on issues relating to both what staff did and how they did it.

Another consideration in relation to our study is the choice of MoHO [42] as theoretical framework for the initial steps of the analysis. It is plausible that other frameworks would have rendered slightly different results, but we were careful to stay close to the data in the inductive steps of the analysis to accurately represent the voices of the older persons. MoHO was chosen based on its ability to facilitate the exploration of what might affect participation, something that has been missing in previous research on and with participatory endeavours. The MoHO concepts were used to explain the nature of performance in relation to participation in research (performance capacity), the routine patterning of everyday life (habituation), the motivation for participation (volition), and the dynamic influence of the institutional culture on participation [42]. This was considered essential to describe what might influence the ability of people living in residential care facilities to participate in research on their own terms. Being initially bound up with disbeliefs in themselves and their own abilities, they trusted staff to take care of the camera on their behalf. Although this involves a risk of a negative impact on their sense of capacity, there were no indications that the photographs would have depicted other situations should the older persons have taken the photographs themselves.

## Conclusion

The major finding of our study is the illustration of how photo-elicitation interviews can be used to minimise the impact of the institutional culture on participatory research in residential care facilities. Thus, a key challenge for participatory research in residential care facilities is to develop dialogues between all people involved to allow for diverging perceptions. With the aim to evaluate photo-elicitation interviews as a participatory research method with persons living in residential care facilities, our study contributed to an awareness of the older persons’ abilities to contribute to the research process. As such, photo-elicitation interviews could be an important tool for participatory research, facilitating co-creation of knowledge, empowerment, and change. Our findings indicate that photo-elicitation interviews are a useful approach for participatory research with people living in residential care facilities. As all other methods, however, it is not without limitations. For instance, getting into the right headspace seems to be a key, and it is important to consider the researcher’s experience and approach when conducting the interviews. Based on our findings, we suggest further explorations on the dynamic relationship between physical, social, and cultural aspects of residential care facilities. Enquiring how these might influence the choice and opportunities for people living in residential care facilities to participate in research are key to designing participatory research projects in collaboration with all people involved.

## Data Availability

The datasets generated and analysed during the current study are not publicly available due to the information provided to the involved persons when obtaining their informed consent, stating that all attempts would be made to maintain their confidentiality. De-identified data are available upon reasonable request to enable review and will be stored for 10 years from publication at the University of Gothenburg, Sweden. All data are covered by the Swedish Public Access to Information and Secrecy Act (offentlighets- och sekretesslagen) and a confidentiality assessment (sekretessprövning) will be performed at each individual request. Permission from the University of Gothenburg, the Institute of Neuroscience and Physiology, must be obtained before data can be accessed.

## Abbreviations

WHO: World Health Organization
MoHO: Model of Human Occupation
